# Supplementary material for: Mixtures of large-scale dynamic functional brain network modes
Source: Neuroimage. Author manuscript; Available in PMC 2026 Mar 28. (PMC7618940; doi:10.1016/j.neuroimage.2022.119595)
Supplement: Supplementary Data S1 [file EMS212883-supplement-Supplementary_Data_S1.pdf]

1 Supplementary Information (SI)

1.1 Derivation of the Loss Function

In variational Bayesian inference we infer a parameter by minimising the variational free energy,

$$F = -\int q(\boldsymbol{\theta}_{1:N}|\boldsymbol{x}_{1:N}) \log\left(\frac{p(\boldsymbol{x}_{1:N}|\boldsymbol{\theta}_{1:N})p(\boldsymbol{\theta}_{1:N})}{q(\boldsymbol{\theta}_{1:N}|\boldsymbol{x}_{1:N})}\right) \mathrm{d}\boldsymbol{\theta}_{1:N}. \tag{1}$$

where $q(\boldsymbol{\theta}_{1:N}|\boldsymbol{x}_{1:N})$ is the posterior, $p(\boldsymbol{\theta}_{1:N})$ is the prior and $p(\boldsymbol{x}_{1:N}|\boldsymbol{\theta}_{1:N})$ is the likelihood, $\boldsymbol{\theta}_t$ is the logit at each time point, $\boldsymbol{x}_t$ is the observed data at each time point and $t = 1, ..., N$ denotes the time index. We can separate the logarithm into two terms,

$$\begin{aligned} F = &-\int q(\boldsymbol{\theta}_{1:N}|\boldsymbol{x}_{1:N}) \log\left(p(\boldsymbol{x}_{1:N}|\boldsymbol{\theta}_{1:N})\right) \mathrm{d}\boldsymbol{\theta}_{1:N} \\ &+ \int q(\boldsymbol{\theta}_{1:N}|\boldsymbol{x}_{1:N}) \log\left(\frac{q(\boldsymbol{\theta}_{1:N}|\boldsymbol{x}_{1:N})}{p(\boldsymbol{\theta}_{1:N})}\right) \mathrm{d}\boldsymbol{\theta}_{1:N}. \\ = &-\mathrm{LL} + \mathrm{KL}. \end{aligned} \tag{2}$$

LL is referred to as the *log-likelihood term* and KL is referred to as the *KL divergence term.*

Considering the log-likelihood term,

$$\mathrm{LL} = \int q(\boldsymbol{\theta}_{1:N}|\boldsymbol{x}_{1:N}) \log\left(p(\boldsymbol{x}_{1:N}|\boldsymbol{\theta}_{1:N})\right) \mathrm{d}\boldsymbol{\theta}_{1:N}. \tag{3}$$

We use the mean field approximation for the posterior,

$$q(\boldsymbol{\theta}_{1:N}|\boldsymbol{x}_{1:N}) = \prod_{t=1}^{N} q(\boldsymbol{\theta}_t|\boldsymbol{x}_{1:N}). \tag{4}$$

Each factor $q(\boldsymbol{\theta}_t|\boldsymbol{x}_{1:N})$ is a multivariate normal distribution parameterised by a mean vector $\boldsymbol{m}_{\theta_t}(\boldsymbol{x}_{1:N})$ and diagonal covariance matrix $\boldsymbol{s}^2_{\theta_t}(\boldsymbol{x}_{1:N})$, i.e.

$$q(\boldsymbol{\theta}_t|\boldsymbol{x}_{1:N}) = \mathcal{N}(\boldsymbol{m}_{\theta_t}(\boldsymbol{x}_{1:N}), \boldsymbol{s}^2_{\theta_t}(\boldsymbol{x}_{1:N})) \tag{5}$$

We also assume the data at each time point is independent and only depends on the logit at that time point, i.e. we factorise the likelihood as

$$p(\boldsymbol{x}_{1:N}|\boldsymbol{\theta}_{1:N}) = \prod_{t=1}^{N} p(\boldsymbol{x}_t|\boldsymbol{\theta}_t). \tag{6}$$

We assume a multivariate normal distribution for the data, i.e.

$$p(\boldsymbol{x}_t|\boldsymbol{\theta}_t) = \mathcal{N}(\boldsymbol{m}(\boldsymbol{\theta}_t), \boldsymbol{C}(\boldsymbol{\theta}_t)) \tag{7}$$

Substituting Equations (4) and (6) into Equation (3),

$$\begin{aligned} \mathrm{LL} &= \int \left[\prod_{\tau=1}^{N} q(\boldsymbol{\theta}_\tau|\boldsymbol{x}_{1:N})\right] \log\left(\prod_{t=1}^{N} p(\boldsymbol{x}_t|\boldsymbol{\theta}_t)\right) \mathrm{d}\boldsymbol{\theta}_{1:N} \\ &= \sum_{t=1}^{N} \int \left[\prod_{\tau=1}^{N} q(\boldsymbol{\theta}_\tau|\boldsymbol{x}_{1:N})\right] \log\left(p(\boldsymbol{x}_t|\boldsymbol{\theta}_t)\right) \mathrm{d}\boldsymbol{\theta}_{1:N} \end{aligned} \tag{8}$$

For each term in the summation we can factorise the integral as

$$\begin{aligned}\mathrm{LL} &= \sum_{t=1}^{N}\left[\int q(\boldsymbol{\theta}_t|\boldsymbol{x}_t)\log\left(p(\boldsymbol{x}_t|\boldsymbol{\theta}_t)\right)\mathrm{d}\boldsymbol{\theta}_t \prod_{\tau=1,\tau\neq t}^{N}\int q(\boldsymbol{\theta}_\tau|\boldsymbol{x}_{1:N})\mathrm{d}\boldsymbol{\theta}_\tau\right] \\ &= \sum_{t=1}^{N}\int q(\boldsymbol{\theta}_t|\boldsymbol{x}_t)\log\left(p(\boldsymbol{x}_t|\boldsymbol{\theta}_t)\right)\mathrm{d}\boldsymbol{\theta}_t.\end{aligned} \tag{9}$$

We can use a Monte Carlo estimate to calculate this as

$$\mathrm{LL} \approx \sum_{t=1}^{N}\frac{1}{M}\sum_{s=1}^{M}\log\left(p(\boldsymbol{x}_t|\boldsymbol{\theta}_t^s)\right), \tag{10}$$

where $\boldsymbol{\theta}_t^s$ denotes the sth sample from the posterior distribution $q(\boldsymbol{\theta}_t|\boldsymbol{x}_{1:N})$ at time point t. In practice we use just one sample, i.e. $M = 1$. Therefore, the log-likelihood term is approximated by

$$\mathrm{LL} \approx \sum_{t=1}^{N}\log\left(p(\boldsymbol{x}_t|\boldsymbol{\theta}_t^1)\right). \tag{11}$$

Considering the KL divergence term,

$$\mathrm{KL} = \int q(\boldsymbol{\theta}_{1:N}|\boldsymbol{x}_{1:N})\log\left(\frac{q(\boldsymbol{\theta}_{1:N}|\boldsymbol{x}_{1:N})}{p(\boldsymbol{\theta}_{1:N})}\right)\mathrm{d}\boldsymbol{\theta}_{1:N}. \tag{12}$$

We use the mean field approximation for the posterior (Equation (4)) and factorise the prior as

$$p(\boldsymbol{\theta}_{1:N}) = p(\boldsymbol{\theta}_1)\prod_{t=2}^{N}p(\boldsymbol{\theta}_t|\boldsymbol{\theta}_{1:t-1}). \tag{13}$$

With these substitutions the KL divergence term becomes

$$\mathrm{KL} = \int\left[\prod_{\tau=1}^{N}q(\boldsymbol{\theta}_\tau|\boldsymbol{x}_{1:N})\right]\log\left(\frac{\prod_{t=1}^{N}q(\boldsymbol{\theta}_t|\boldsymbol{x}_{1:N})}{p(\boldsymbol{\theta}_1)\prod_{\kappa=2}^{N}p(\boldsymbol{\theta}_\kappa|\boldsymbol{\theta}_{1:\kappa-1})}\right)\mathrm{d}\boldsymbol{\theta}_{1:N}. \tag{14}$$

We split up the logarithm as

$$\mathrm{KL} = \int\left[\prod_{\tau=1}^{N}q(\boldsymbol{\theta}_\tau|\boldsymbol{x}_{1:N})\right]\left[\log\left(\frac{q(\boldsymbol{\theta}_1|\boldsymbol{x}_1)}{p(\boldsymbol{\theta}_1|\boldsymbol{x}_1)}\right)+\log\left(\prod_{t=2}^{N}\frac{q(\boldsymbol{\theta}_t|\boldsymbol{x}_{1:N})}{p(\boldsymbol{\theta}_t|\boldsymbol{\theta}_{1:t-1})}\right)\right]\mathrm{d}\boldsymbol{\theta}_{1:N} \tag{15}$$

and clip the first logarithm to give

$$\begin{aligned}\mathrm{KL} &\approx \int\left[\prod_{\tau=1}^{N}q(\boldsymbol{\theta}_\tau|\boldsymbol{x}_{1:N})\right]\log\left(\prod_{t=2}^{N}\frac{q(\boldsymbol{\theta}_t|\boldsymbol{x}_{1:N})}{p(\boldsymbol{\theta}_t|\boldsymbol{\theta}_{1:t-1})}\right)\mathrm{d}\boldsymbol{\theta}_{1:N} \\ &\approx \sum_{t=2}^{N}\int\left[\prod_{\tau=1}^{N}q(\boldsymbol{\theta}_\tau|\boldsymbol{x}_{1:N})\right]\log\left(\frac{q(\boldsymbol{\theta}_t|\boldsymbol{x}_{1:N})}{p(\boldsymbol{\theta}_{1:N}|\boldsymbol{\theta}_{1:t-1})}\right)\mathrm{d}\boldsymbol{\theta}_{1:N}\end{aligned} \tag{16}$$

For each term in the summation, we can factorise the integral as

$$\begin{aligned}\text{KL} &\approx \sum_{t=2}^{N} \int \left[\prod_{\tau=1}^{t} q(\boldsymbol{\theta}_\tau|\boldsymbol{x}_{1:N})\right] \log\left(\frac{q(\boldsymbol{\theta}_t|\boldsymbol{x}_{1:N})}{p(\boldsymbol{\theta}_t|\boldsymbol{\theta}_{1:t-1})}\right) \mathrm{d}\boldsymbol{\theta}_{1:t} \left[\prod_{\kappa=t+1}^{N} \int q(\boldsymbol{\theta}_\kappa|\boldsymbol{x}_{1:N}) \mathrm{d}\boldsymbol{\theta}_k\right] \\ &\approx \sum_{t=2}^{N} \int \left[\prod_{\tau=1}^{t} q(\boldsymbol{\theta}_\tau|\boldsymbol{x}_{1:N})\right] \log\left(\frac{q(\boldsymbol{\theta}_t|\boldsymbol{x}_{1:N})}{p(\boldsymbol{\theta}_t|\boldsymbol{\theta}_{1:t-1})}\right) \mathrm{d}\boldsymbol{\theta}_{1:t}\end{aligned} \tag{17}$$

We denote the integral over $\mathrm{d}\boldsymbol{\theta}_t$ by

$$D_{\text{KL}}\left(q(\boldsymbol{\theta}_t|\boldsymbol{x}_{1:N}) \mid\mid p(\boldsymbol{\theta}_t|\boldsymbol{\theta}_{1:t-1})\right) = \int q(\boldsymbol{\theta}_t|\boldsymbol{x}_{1:N}) \log\left(\frac{q(\boldsymbol{\theta}_t|\boldsymbol{x}_{1:N})}{p(\boldsymbol{\theta}_t|\boldsymbol{\theta}_{1:t-1})}\right) \mathrm{d}\boldsymbol{\theta}_t. \tag{18}$$

Substituting this into the KL divergence term, we get

$$\text{KL} \approx \sum_{t=2}^{N} \int \prod_{\tau=1}^{t-1} q(\boldsymbol{\theta}_\tau|\boldsymbol{x}_{1:N}) D_{\text{KL}}\left(q(\boldsymbol{\theta}_t|\boldsymbol{x}_{1:N}) \mid\mid p(\boldsymbol{\theta}_t|\boldsymbol{\theta}_{1:t-1})\right) \mathrm{d}\boldsymbol{\theta}_{1:t-1} \tag{19}$$

We use a Monte Carlo estimate using a single sample from each posteriors $q(\boldsymbol{\theta}_1|\boldsymbol{x}_{1:N}), ..., q(\boldsymbol{\theta}_{t-1}|\boldsymbol{x}_{1:N})$. Therefore, our KL divergence term is

$$\text{KL} \approx \sum_{t=2}^{N} D_{\text{KL}}\left(q(\boldsymbol{\theta}_t|\boldsymbol{x}_{1:N})) \mid\mid p(\boldsymbol{\theta}_t|\boldsymbol{\theta}^1_{1:t-1})\right). \tag{20}$$

The prior $p(\boldsymbol{\theta}_t|\boldsymbol{\theta}^1_{1:t-1})$ is a multivariate normal distribution parameterised by a mean vector $\boldsymbol{\mu}_{\theta_t}(\boldsymbol{\theta}^1_{1:t-1})$ and diagonal covariance matrix $\boldsymbol{\sigma}^2_{\theta_t}(\boldsymbol{\theta}^1_{1:t-1})$, i.e.

$$p(\boldsymbol{\theta}_t|\boldsymbol{\theta}^1_{1:t-1}) = \mathcal{N}(\mu_{\theta_t}(\boldsymbol{\theta}^1_{1:t-1}), \boldsymbol{\sigma}^2_{\theta_t}(\boldsymbol{\theta}^1_{1:t-1})). \tag{21}$$

The parameters $\boldsymbol{\mu}_{\theta_t}(\boldsymbol{\theta}^1_{1:t-1})$ and $\boldsymbol{\sigma}^2_{\theta_t}(\boldsymbol{\theta}^1_{1:t-1})$ are calculated using the model RNN.

We use stochastic gradient descent to minimise a loss function. The loss function we use is

$$\begin{aligned}\mathcal{L} &= F = -\text{LL} + \text{KL} \\ \mathcal{L} &= -\sum_{t=1}^{N} \log\left(p(\boldsymbol{x}_t|\boldsymbol{\theta}^1_t)\right) + \sum_{t=2}^{N} D_{\text{KL}}\left(q(\boldsymbol{\theta}_t|\boldsymbol{x}_{1:N}) \mid\mid p(\boldsymbol{\theta}_t|\boldsymbol{\theta}^1_{1:t-1})\right).\end{aligned} \tag{22}$$

Using this loss function will minimise the variational free energy, or equivalently, it will maximise the evidence lower bound [1].

1.2 Training and Hyperparameters

Before training the model we prepare the source reconstructed data. We applied time-delay embedding, PCA and standardisation. Time-delay embedding and PCA are summarised in Figure S1. The procedure used to train the model and choices for hyperparameters are discussed below.

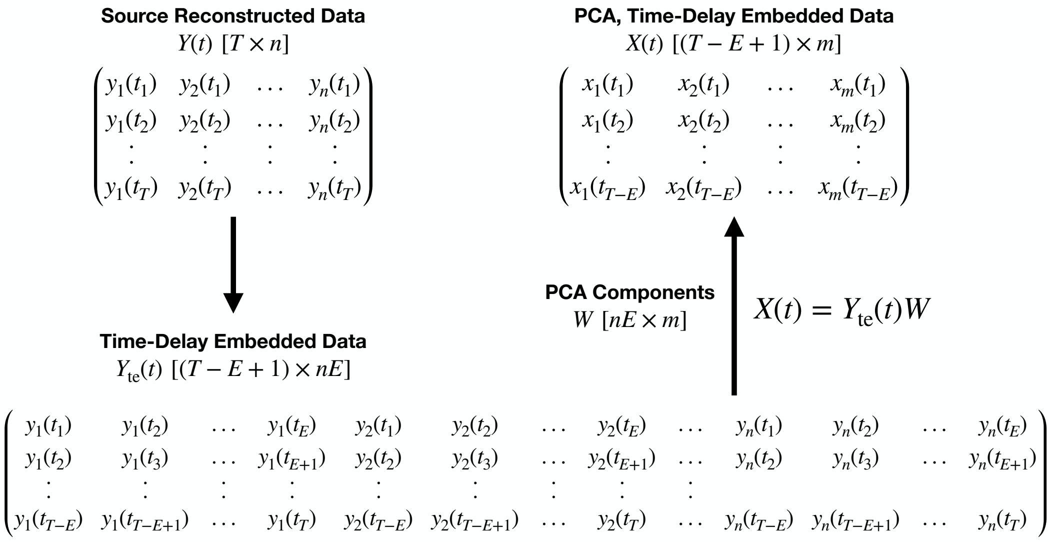

Figure S1: Preparation applied to the source reconstructed data before training. The use of time-delay embedding encodes spectral properties of the training data into the covariance by adding extra elements that correspond to the auto-correlation function to the matrix. PCA is performed to reduce the number of channels so that the data is not too large to fit within GPU memory. Standardisation is also applied after PCA. T is the number of time points, n is the number of parcels/regions of interest, E is the number of time-delay embeddings and m is the number of channels after PCA.

Logit activation function. To calculate the mixing coefficients from the logits we use a softmax function,

$$\alpha_{jt} = \{\zeta(\boldsymbol{\theta}_t)\}_j = \frac{\exp\left(\frac{\theta_{jt}}{\tau}\right)}{\sum_{j=1}^{J} \exp\left(\frac{\theta_{jt}}{\tau}\right)}, \tag{23}$$

where τ is a hyperparameter known as the temperature, discussed further below. The use of a softmax function imposes the constraint that the α_{jt}-values are positive and the sum of α_{jt} over j is equal to one.

Alpha temperature. We can specify a temperature τ for the softmax activation function. The temperature determines the amount of mixing between modes. A high temperature corresponds to a more even mixture, whereas a low temperature leads to more mutually exclusive modes. In this work, we allow the temperature to be a learnable parameter.

Learning rate. In this work, we use the Adam optimiser [2] to update trainable parameters. The learning rate η is a key hyperparameter of the Adam optimiser, which can affect training. Using a value that is too high can lead to divergence in the loss function, alternatively, one that is too small can lead to slow convergence.

Sequence length. The input to the model is a sequence of data points $\boldsymbol{x}_{1:N}$, where N is the length of the sequence. The sequence length determines the number of previous time

points the model has access to. Therefore, we would like to use the longest sequence length possible. However, the sequence length is limited by the GPU memory that is available and the ability to train the model in a reasonable time frame.

Batch learning. Stochastic gradient descent is performed by estimating the loss for a small group of sequences, referred to as a *batch*. This loss is used to update the trainable parameters before the loss for a new batch is calculated. The number of sequences in a batch is referred to as the *batch size*. We calculate the loss for a batch by averaging the loss for each sequence in the batch. When performing batch learning it is important to shuffle the ordering of the sequences and batches. We did this by first separating the entire dataset into sequences, shuffling the order of the sequences, then grouping sequences in batches and performing one final random reordering to give the training dataset. One training loop through all of the batches is referred to as an *epoch*. The batches are not reshuffled between epochs.

Hidden units and number of LSTM layers. When using an LSTM we must specify a number of hidden units. We found small networks had more stable training than large networks, so only used 64 units in the model and inference LSTM. We also found stacking multiple LSTMs did not improve the model, so in this work we only use one LSTM layer.

Dropout [3]. This is well-known technique used when training a neural network to mitigate overfitting. The use of dropout was found not to benefit the model so no dropout layers have been used in this work.

Normalisation layers [4, 5]. Normalisation layers are often used to train deep neural networks because they help alleviate the the vanishing/exploding gradient problem [6]. In this work, we include a single Layer Normalisation [5] transformation to the output of the LSTMs in Equations (2) and (6) before the affine transformation.

Gradient clipping. RNNs can suffer from exploding gradients when backpropagation occurs through each time step. A strategy proposed in [7] to avoid this is gradient clipping, where we rescale the gradients so that their norm is a particular value when gradient norm would otherwise exceed this value. This strategy has been shown to improve training stability. In this work, we use gradient clipping when training on real MEG data.

Trainable parameters and initialisation. The trainable parameters in this model are:

- The weights and biases of the model and inference LSTM. At the start of training these are set randomly using Glorot initialisation [8].
- Layer Normalisation weights. The output of Layer Normalisation is centred around a learnable β-parameter and scaled with a γ-parameter [9, 5], these parameters were initialised using zeros and ones respectively.
- Dense layer weights and biases for the affine transformations. Glorot initialisation [8] was used for these parameters.
- The alpha temperature. This was initialised using a value of one.
- Elements of the mode means and covariances. In this work, we use zero vectors for the means and an identity matrix for the covariances. When training on MEG data we found the initialisation of the mode covariances to be important. We propose a strategy of initially training the model on the data for a randomly selected single subject to

estimate its covariances. The covariances are initialised with the identity matrix when training on a single subject. This removes the subject-to-subject variability in the data. Then, the model can be trained on the full dataset initialising with the single-subject mode covariances. We found this strategy helped to avoid local optima when learning a high latent dimensionality (e.g. $J > 10$).

Multi-start training. We find the model is sensitive to the initialisation of the trainable parameters, in particular the inference and model RNNs. When training on real MEG data, we observe that the model can converge to different local optima with the same dataset. To help find the global optimum, we propose a multi-start approach, where we train the model for a small number of epochs a few times and performing the full training on the model with the lowest loss at the end of the initial training period. This procedure was used when training on real MEG data and was found to reduce the run-to-run variability.

KL annealing [10] is a technique used at the start of training. An annealing factor λ is introduced into the loss function,

$$\mathcal{L} = -\mathrm{LL} + \lambda \cdot \mathrm{KL}. \tag{24}$$

The annealing factor is a smoothly varying function of the number of training epochs. It takes a value between zero and one. The function used to calculate the annealing factor in this work is

$$\lambda = \frac{1}{2} \tanh\left(\frac{A_S(n_{\mathrm{E}} - \frac{1}{2} n_{\mathrm{AE}})}{n_{\mathrm{AE}}}\right) + \frac{1}{2}, \tag{25}$$

where A_S is the annealing sharpness, which determines the shape of the annealing curve, n_{E} is the number of training epochs and n_{AE} is the number of annealing epochs.

At the start of training, the weights and biases of the model RNN are initialised with samples from a uniform distribution (Glorot initialisation [8]). As a consequence, the model RNN is prone to giving naive outputs/estimates for the logit time course in these early epochs. KL annealing with λ close to zero helps in this period because it prevents the model RNN from influencing the inference RNN before it has learnt a logit time course that is useful for describing the training data. As training progresses, λ tends to one and the model RNN learns the temporal dynamics in the latent representation by predicting probability distribution of the next logit $\boldsymbol{\theta}_t$ from the samples of previous logits $\boldsymbol{\theta}_{1:t-1}$. By doing this, it also regularises the inferred logits for the training data.

1.3 Post-hoc Analysis of Learnt Latent Variables

Once trained, DyNeMo provides us with a mixing coefficient time series for each mode, α_{jt}. We use the inferred mixing coefficients with the source reconstructed data (before preparation) to perform post-hoc analysis. We describe the quantities calculated in our post-hoc analysis below.

Summary statistics. We can summarise the mixing coefficients with statistics, which can give a high-level description of the data. We can take inspiration from the Viterbi path (referred to as the *state time course*) of an HMM[1] [1]. We typically summarise this

[1]The Viterbi path is the maximum a posteriori probability estimate for the state in an HMM.

with state lifetimes[2], interval times and the fractional occupancies [11]. One benefit of the mutual exclusivity assumption made by the HMM is that there are well defined time points when a state is active, making determining a lifetime and interval time straightforward. Contrastingly, DyNeMo provides a description where multiple modes are simultaneously present at each time point. To define when a mode is active we fit a two-component GMM to the mixing coefficient time series of each mode. One of the Gaussian components corresponds to time points when the mode is active whereas the other component corresponds to time points when the mode is inactive. This GMM therefore gives us a mode activation time course, which we can use to compute the usual summary statistics we would calculate with a state time course. Note, we fit a GMM separately to each mode, which enables the possibility of there being time points where multiple modes or no modes activate. The mixing coefficients have a sum to one constraint, which means their distribution is non-Gaussian. Therefore, we transform the data using the logit function[3] and standardise before fitting the GMM to make the distribution more Gaussian. We found defining activations with a GMM led to more stable summary statistics for each mode compared to a simpler approach such as using an argmax operation. This is because with an argmax operation the mode with the largest mixing coefficient depends on the full set of modes, whereas with the GMM approach, each mode is studied in isolation.

Mode spectral properties. Neuronal activity in the brain has oscillatory dynamics. A useful quantity for examining these oscillations is the power spectral density (PSD), which displays the power at each frequency for a given channel (i.e. region of interest). Additionally, the cross spectral density, which displays the power coupling across two channels, is of interest. We can calculate power and cross spectra for each mode directly from source reconstructed data once we have inferred the mixing coefficients.[4] Equation (6) defines a linear mixture of mode covariances. A property of this model is that the PSD of each mode mixes with the same coefficients. We can exploit this property to estimate the spectral properties of each mode. We do this by first calculating a cross spectrogram using the dataset and fitting a linear regression model using the mixing coefficients:

$$\boldsymbol{P}_t(f) = \sum_{j=1}^{J} \alpha_{jt} \boldsymbol{P}_j(f) + \boldsymbol{P}_0(f) + \epsilon_t(f), \tag{26}$$

where $\boldsymbol{P}_t(f)$ is the cross spectrogram of each pair of channels, $\boldsymbol{P}_j(f)$ are regression coefficients, which are our mode cross spectra, $\boldsymbol{P}_0(f)$ is a mean term, $\epsilon_t(f)$ is a residual and f is the frequency. We standardise (z-transform) the mixing coefficients across the time dimension before calculating the linear regression. This results in the mean term $\boldsymbol{P}_0(f)$ corresponding to the time-averaged PSD and the regression coefficients $\boldsymbol{P}_j(f)$ corresponding

[2] Also known as the dwell time.

[3] $\text{logit}(x) = \ln\left(\frac{x}{1-x}\right)$.

[4] It is possible to extract mode PSDs from the mode covariance matrices by reversing the PCA, which gives the auto-covariance of the time-delay embedded data. This matrix contains an estimate of the autocorrelation function, which can be Fourier transformed to give a mode PSD. However, the resolution of this PSD is limited by the number of time-delay embeddings, which results in a low-resolution PSD. This is why estimating the mode PSD using the inferred mixing coefficients and source reconstructed data is preferred.

to PSDs relative to the time-averaged PSD. We calculate the cross spectrogram with the source reconstructed data using Welch's method [12], which involves segmenting the data into overlapping windows and performing a Fourier transform:

$$P_{mn,t}(f) = \frac{1}{f_s W^2} X_{m,t}(f) X^*_{n,t}(f), \tag{27}$$

where m and n are channels, f_s is the sampling frequency, W is the number of samples in the window, $X_{m,t}(f)$ is the Fourier transform of the data in a window centred at time t and $*$ denotes the complex conjugate.

Mode power maps. Additionally, the spatial distribution of power across the brain is of interest. We can examine the spatial power distribution of each mode separately, which can highlight the areas of the brain that are active for each mode. Using the cross spectrogram regression method (Equation (26)), we obtain a PSD for each channel for a given mode. As each channel corresponds to a region of interest, we obtain a PSD for the activity at each region of interest. The integral of a PSD is the power. Plotting the power at each region of interest as a two-dimensional heat map projected onto the surface of the brain shows the spatial distribution of power for a given mode. It is often more interesting to plot the power relative to a reference rather than the absolute value to highlight differences in the power maps of each mode. In this work, we calculate the power by integrating the mode PSDs $\boldsymbol{P}_j(f)$ without the mean term $\boldsymbol{P}_0(f)$. This gives us the power distribution relative to the mean power common to all modes. We also subtract the mean power across modes for each region of interest separately when displaying the power maps to help highlight relative differences between the modes. No thresholding is applied to the power maps. Additionally, the surface plotting function we use interpolates the power between parcels for visualisation.

Mode FC maps. In addition to power maps, FC maps reveal the brain networks that are present for each mode. We use the coherence as our measure of FC, which quantifies the stability of phase difference between two regions of interest, thereby providing a measure of synchronisation or phase-locking between brain regions. We choose this measure because it gives us a direct estimate of oscillatory synchronicity, which has been proposed as a mechanism for neuronal communication [13]. To calculate the coherence we use the mode cross spectra estimated with the linear regression model in Equation (26). Estimating the coherence for a window requires us to average multiple estimates of the cross spectra within that window. We do this by dividing each window into a set of sub-windows and taking the average cross spectra for each sub-window. Using the cross spectra we calculate a coherence for each pair of channels. However, most of these connections correspond to a background level of activity that is are common to all modes. To identify the most prominent connections, we fit a two-component GMM to the distribution of coherences relative to the mean. We standardise the relative coherence values before fitting the GMM. One component of the GMM corresponds to a population of background coherence, whereas the other corresponds to prominently high relative coherence values. We plot the connection edges in the original coherence matrix that correspond to high relative coherence values. If we are unable to identify two components, we plot the top 5% of connections.

To calculate power and FC maps, we use a window length of 4 s, sub-window length of 0.5 s, window step size of 0.08 s, and apply a Hann windowing function to each sub-window before performing the Fourier transform to calculate the cross spectrogram. We calculate the cross spectrogram and perform the linear regression separately for each subject. We then average the subject-specific mode PSDs and coherences over subjects to obtain group-level spectra. We also apply a Hann function to sub-windows of the mixing coefficient time series to match the windowing applied to the data and average the values across a full window to calculate the α_{jt} value to use in the regression. To calculate the spectrograms used to the study the evoked response to task (Section 3.4) we use a window length of 0.5 s and do not separate the window into sub-windows.

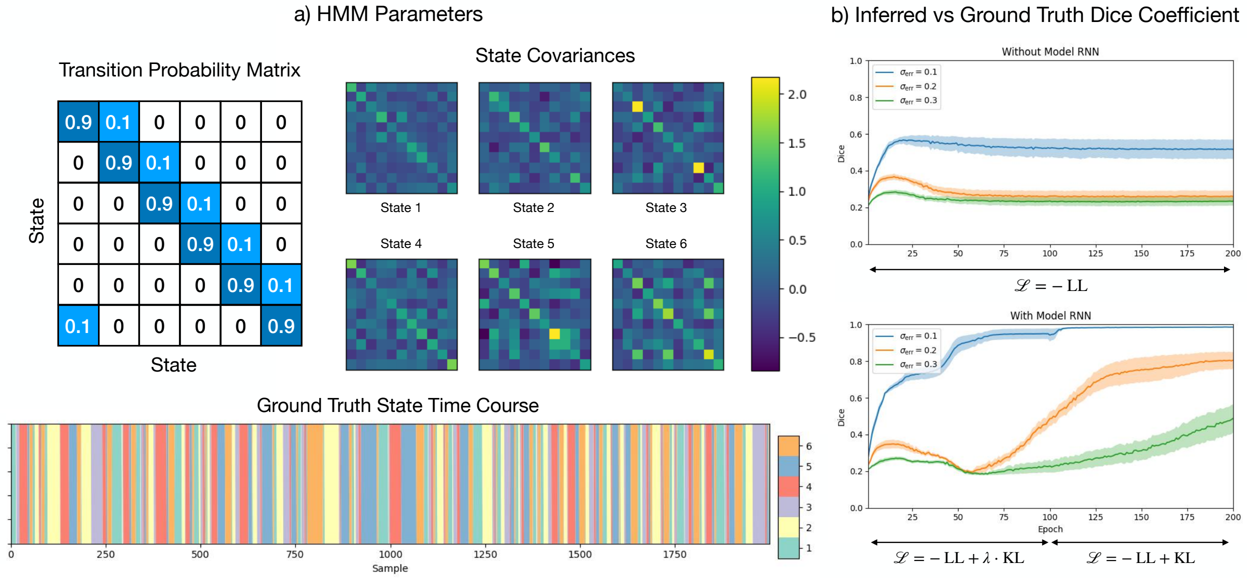

Figure S2: Temporal regularisation from the model RNN improves inference. a) HMM parameters used to simulate data. A 6 state, 11 channel HMM was simulated with the transition probability matrix shown. 25,600 samples were generated. Only the first 2,000 time steps of the state time course are shown. b) Dice vs epoch when minimising the negative log-likelihood loss only (top) and the variational free energy (bottom) for different added noise, σ_{err}, which is the standard deviation of normally distributed errors added to each channel at each time point. $\mathcal{L}$ denotes the loss function used for each stage of training (see Eqs. (24) and (25)). Each model was fitted 5 times to the same dataset. The solid line is the mean and the shaded area shows the standard error on the mean.

1.4 Regularisation from the Model RNN

It is possible to construct a model for the training data without including the model RNN. For example, we could construct a standard variational autoencoder [14], where a normal distribution with zero mean and unit variance takes the place of the model RNN (i.e. the prior). Alternatively, point estimates for logits could be learnt and no prior would be needed. So why include a model RNN? There are two reasons, firstly learning a generative model

in itself is useful, and secondly the model RNN regularises the inferred logits and can help alleviate overfitting to noise in the training dataset. The latter can be demonstrated by training DyNeMo on simulated HMM data with varying noise added. Parameters of the HMM simulation are shown in Figure S2a. We measure the inference accuracy of the model by calculating the dice coefficient of the inferred state time course and the ground truth from the simulation. Figure S2b shows the dice vs epoch during training for different levels of noise. The top figure is the achieved dice coefficient when minimising the negative log-likelihood loss only ($\mathcal{L} = -\mathrm{LL}$). When the noise is high the model struggles to correctly infer the ground truth state time course. The bottom figure is the achieved dice when minimising the variational free energy ($\mathcal{L} = -\mathrm{LL} + \lambda \cdot \mathrm{KL}$). KL annealing was applied during training for the first 100 epochs. The influence of the model RNN starts to appear when λ is sufficiently large, which is after the 50th epoch roughly. Figure S2 shows the inclusion of the model RNN leads to a better inference when training on noisy data.

1.5 Run-to-Run Variability and Number of Modes

When training DyNeMo, our objective is to the minimise the loss function (variational free energy) by updating the trainable parameters of the model. Due to the random initialisation of the RNN weights and the use of stochastic gradient descent to update the parameters, it is possible for DyNeMo to converge to different local optima in the loss function. Neuroimaging data itself is complex and it is reasonable to expect there could be multiple models with different parameters that can lead to similar loss values. In this section we look at the variability in the final loss value for different training runs and look at the impact of varying the number of modes.

First, we look at the simulation datasets. In Bayesian inference the model evidence, which is approximated by the variational free energy [15], can be used to compare models with different hyperparameters. This allows us to select the number of modes to infer using the variational free energy. Figure S3 shows the variational free energy of DyNeMo trained on the simulation datasets described in Sections 2.3.1 and 2.3.2 as a function of number of modes. The ground truth number of modes was 3 for simulation 1 and 6 for simulation 2. We can see the variational free energy decreases until the correct number of modes is reached. It then flattens, we see adding more modes no longer shows an improvement. This indicates the variational free energy can be used to correctly select the number of modes.

Turning to the resting-state MEG dataset, we evaluate the run-to-run variability in the loss. Figure S4 shows the final training and validation loss as a function of number of modes. As the number of modes increases so does the variability in the loss. Our strategy for finding the global optimum involves training DyNeMo multiple times and selecting the model with the lowest loss. Although there's a systematic offset in the validation loss compared to the training loss, which suggests overfitting to the 45 subject dataset, the difference remains the same as the number of modes is increased, which means overfitting does not get worse with more modes. The systematic offset is likely to be due to the fact that the validation dataset contained unseen subjects and the model not generalising to new subjects very well. Over this range, the loss decreases monotonically meaning more modes provide a better description of the data. Unlike the simulation studies, we cannot see a clear minimum in the loss function. This indicates the optimum number of modes maybe greater than 30. However, the aim of

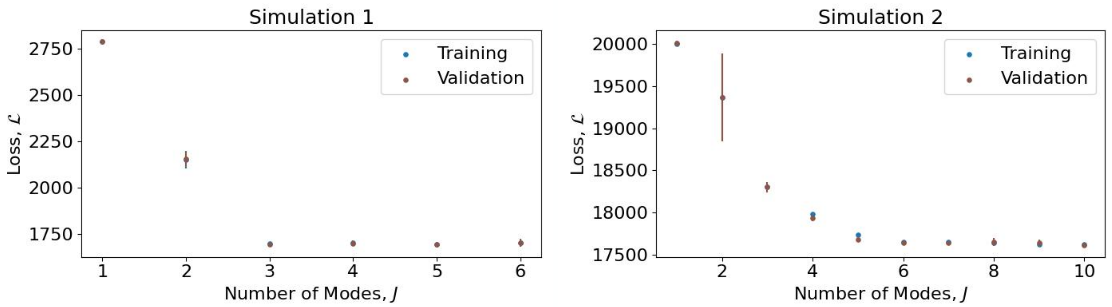

Figure S3: The loss (variational free energy) indicates the optimum number of modes. Simulations 1 and 2 are described in Section 3.1 and 3.2. The ground truth in these simulations was dataset generated using 3 and 6 modes respectively. The mean training (blue) and validation loss (brown) for a batch vs the number of modes. Each data point is the average of five runs. The error bar is one standard deviation. The same training dataset was used here and the results shown in Figures 4 and 5. An additional 5120 data points were simulated for the validation dataset.

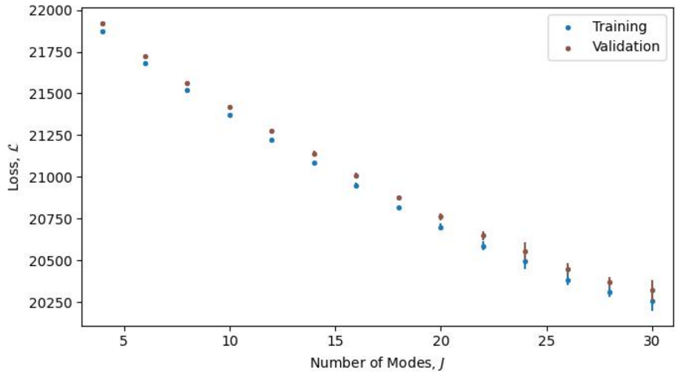

Figure S4: More modes provide a better description of the data. The mean training (blue) and validation loss (brown) for a batch vs the number of modes. We split the resting-state MEG dataset into a 45 subject training dataset and 10 subject validation dataset. Each data point is the average of ten runs. The error bar is one standard deviation. For 25 modes or less the error bar is too small to be seen.

this model is to provide a low-dimensional interpretable description of the data. Therefore, rather than using the variational free energy to determine the number of modes, we preselect a low value as a hyperparameter.

Figure S5 shows the power maps for the run with the best loss when fitting 4-12 modes. As the number of modes increases the activation of each brain region becomes more localised. This is expected as increasing the number of modes allows greater precision in reconstructing the time-varying covariance $\boldsymbol{C}_t$ from the mode covariances $\boldsymbol{D}_j$ (see Equation (4)). When we fit 4 modes, we see power is divided amongst the four lobes: the occipital, parietal, temporal and frontal lobe. As we increase the number of modes, the number of possible ways to distribute power amongst the modes also increases. In this work, we choose to fit 10 modes to MEG data as a trade off between obtaining an interesting description of the data and

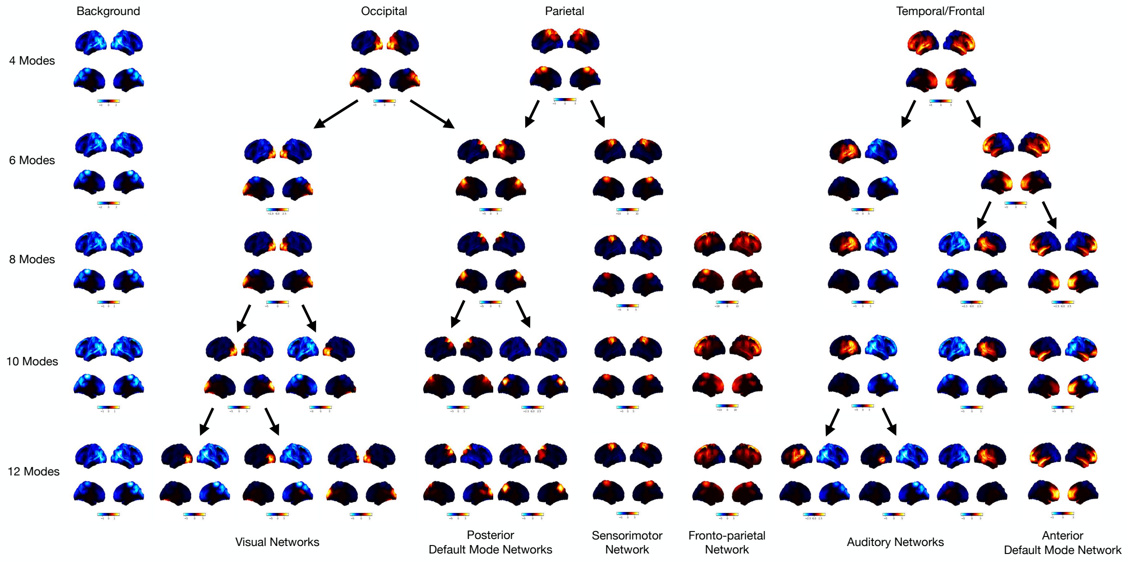

Figure S5: Power maps show more localised activation with an increasing number of modes. These power maps were obtained from a model trained on resting-state MEG data from 45 subjects. Arrows indicate when a power map has split as the number of modes was increased. The top two views on the brain in the power map plots are lateral surfaces and the bottom two are medial surfaces.

minimising the variability in the power maps of each run.

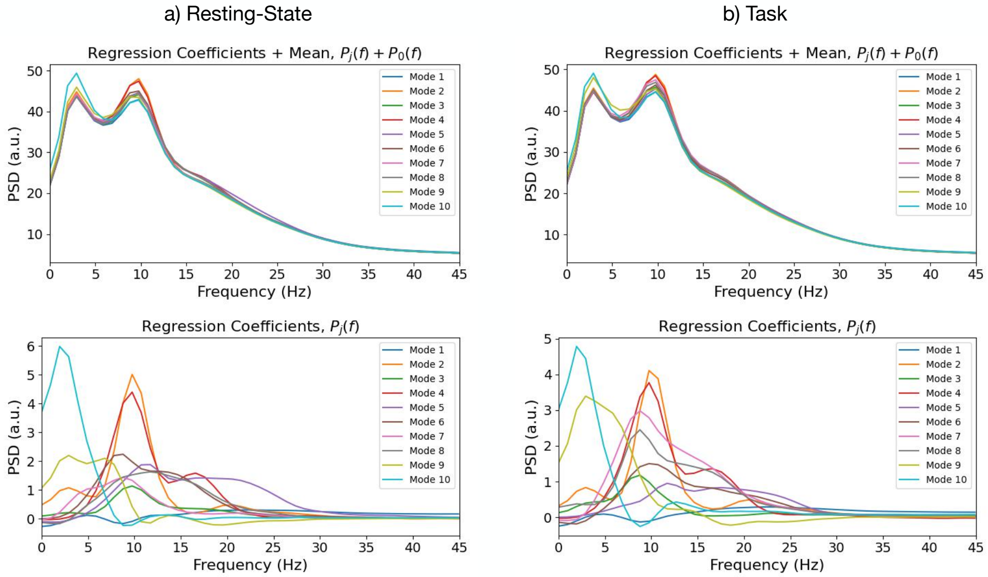

Figure S6: DyNeMo learns spectrally distinct modes. Mode PSDs including the activity common to all modes (top) and mode PSD relative to the mean activity common to all modes (bottom) for the resting-state (a) and task (b) MEG dataset. The mean PSD across channels is shown.

1.6 Mode PSDs

Figure S6 shows the PSD of each mode calculated with the regression method described in Section 2.4 for the resting-state and task MEG dataset. In Figure S6 (top) we see all modes have a PSD with a $1/f$ profile and exhibit a prominent 10 Hz peak. The drop off towards 0 Hz is due to filtering applied during preprocessing. In Figure S6 (bottom) we see the mode PSD relative to the activity shared across modes. We see differences in the PSD of each mode consistent with expected frequency content of activity at locations shown in the corresponding power maps.

1.7 HMMs Trained on the MEG Datasets

The HMM was also fitted to the MEG datasets described in Section 2.3.3 for comparison with DyNeMo. Figure S7 shows the power maps, FC maps and PSDs of the HMM states inferred on the resting-state MEG dataset and Figure S9 shows the power maps, FC maps and PSDs of the HMM states inferred on the task MEG dataset.

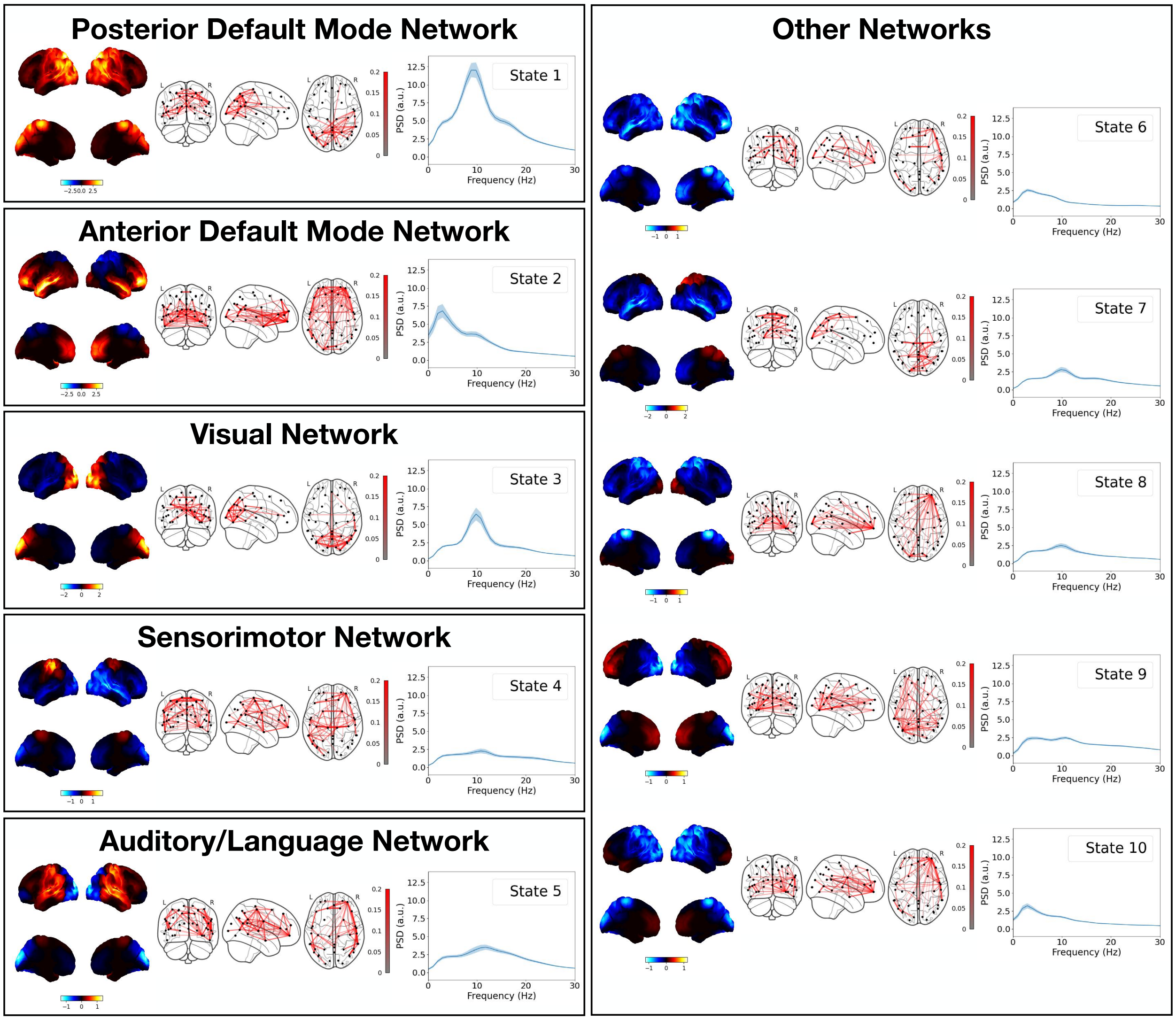

Figure S7: HMM states inferred on a resting-state MEG dataset consisting of 55 subjects. Each box shows the power map (left), FC map (middle) and PSD averaged over regions of interest (right) for each group. The top two views on the brain in the power map plots are lateral surfaces and the bottom two are medial surfaces. The shaded area in the PSD plots shows the standard error on the mean.

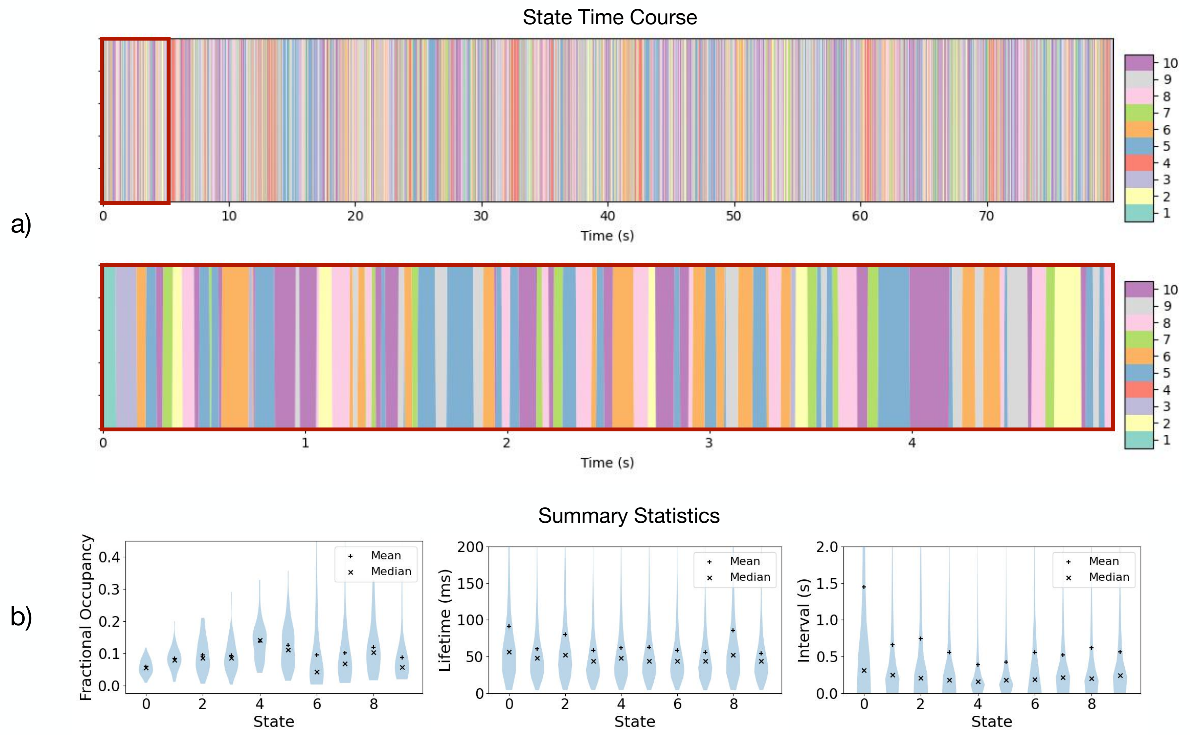

Figure S8: State time course and summary statistics for the HMM fit to the resting-state MEG dataset. a) Inferred state time course for the first 80 s of the first subject (top) and zoomed in on the first 5 s (bottom). b) Distribution of fractional occupancies over subjects and distribution of state lifetimes and intervals for all subjects.

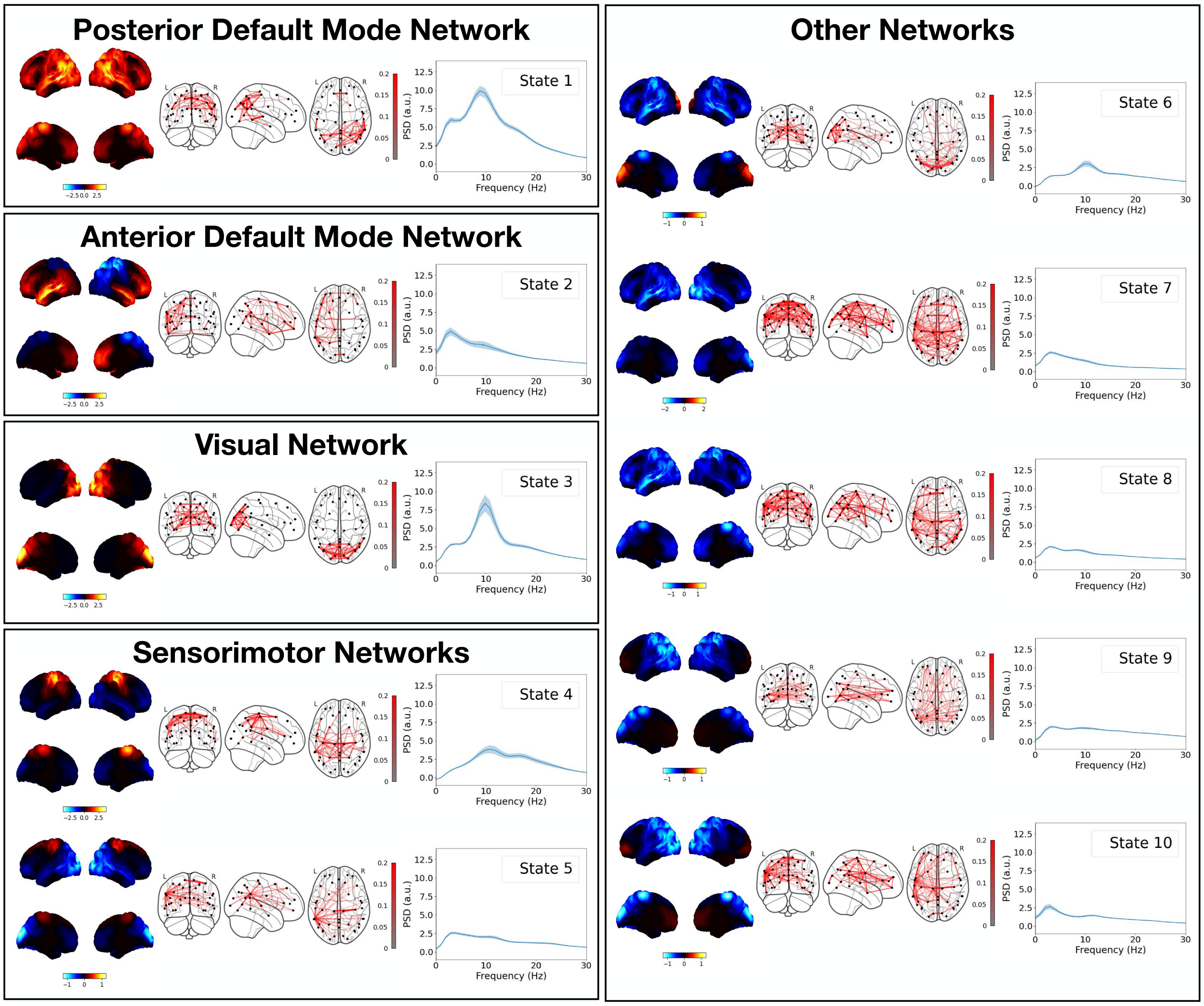

Figure S9: HMM states inferred on a visuomotor task MEG dataset consisting of 51 subjects. Each box shows the power map (left), FC map (middle) and PSD averaged over regions of interest (right) for each group. The top two views on the brain in the power map plots are lateral surfaces and the bottom two are medial surfaces. The shaded area in the PSD plots shows the standard error on the mean.

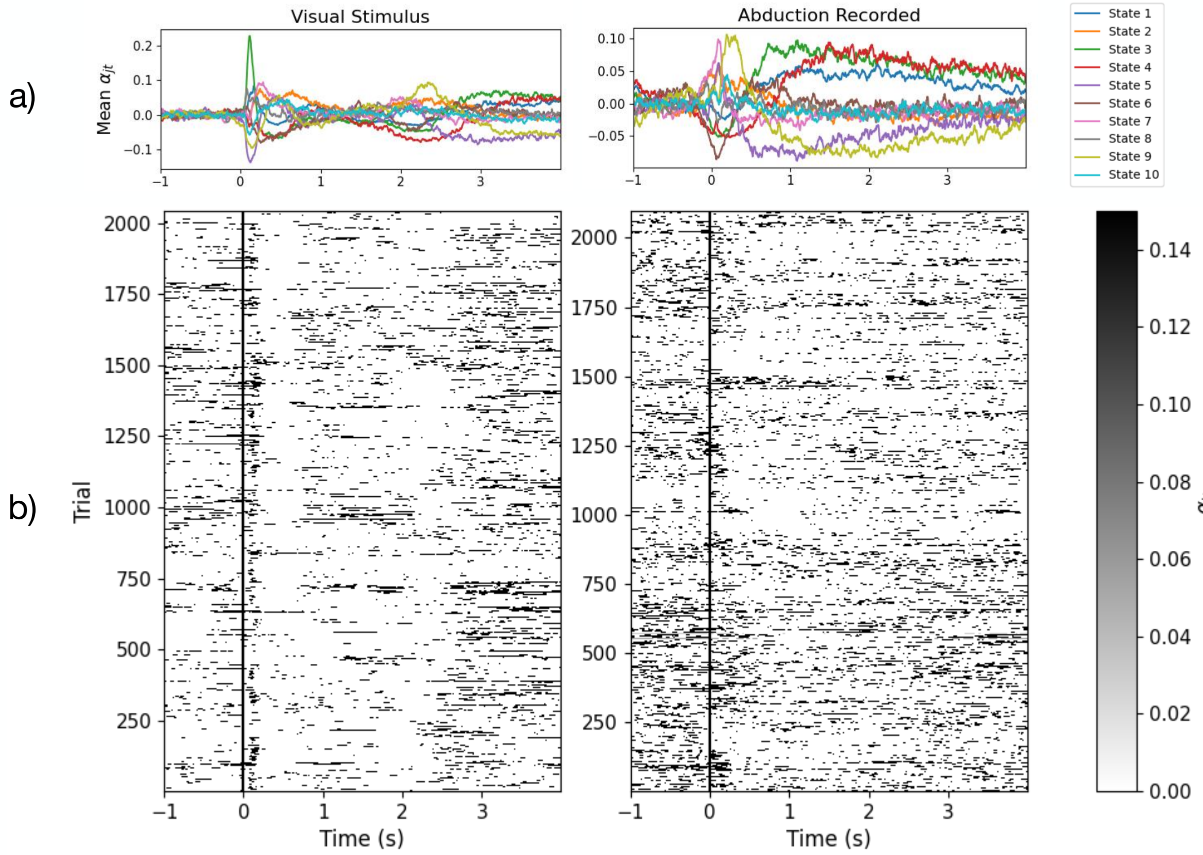

Figure S10: An evoked response to the visuomotor task is seen across trials with the HMM fit to the visuomotor task MEG dataset. a) State time courses epoched around the visual (left) and abduction (right) task. The average over trials is shown. b) Individual trial responses (state time course) for state 3 (visual, left) and state 5 (sensorimotor, right). The visual stimulus/abduction task occurs at Time = 0 s.

References

[1] Bishop, C. Pattern Recognition and Machine Learning. (Springer, 2007).

[2] Kingma, D. and Ba, J. Adam: A method for stochastic optimization. *arXiv preprint arXiv:1412.6980.* (2014).

[3] Srivastava, N., Hinton, G., Krizhevsky, A., Sutskever, I. and Salakhutdinov, R. Dropout: A Simple Way to Prevent Neural Networks from Overfitting. *Journal Of Machine Learning Research.* **15**, 1929-1958 (2014).

[4] Ioffe, S. and Szegedy, C. Batch normalization: accelerating deep network training by reducing internal covariate shift. *International Conference On Machine Learning.* pp. 448-456 (2015).

[5] Ba, J., Kiros, J. and Hinton, G. Layer normalization. *arXiv preprint arXiv:1607.06450.* (2016).

[6] Bengio, Y., Simard, P. and Frasconi, P. Learning long-term dependencies with gradient descent is difficult. *IEEE Trans. Neural Netw.* **5**, 157-166 (1994).

[7] Pascanu, R., Mikolov, T. and Bengio, Y. On the difficulty of training recurrent neural networks. *International Conference On Machine Learning.* pp. 1310-1318 (2013).

[8] Glorot, X. and Bengio, Y. Understanding the difficulty of training deep feedforward neural networks. *Proceedings Of The Thirteenth International Conference On Artificial Intelligence And Statistics.* pp. 249-256 (2010).

[9] TensorFlow: Large-Scale Machine Learning on Heterogeneous Systems, `https://www.tensorflow.org/`.

[10] Bowman, S., Vilnis, L., Vinyals, O., Dai, A., Jozefowicz, R. and Bengio, S. Generating sentences from a continuous space. *arXiv preprint arXiv:1511.06349.* (2015).

[11] Baker, A., Brookes, M., Rezek, I., Smith, S., Behrens, T., Smith, P. and Woolrich, M. Fast transient networks in spontaneous human brain activity. *Elife.* **3** pp. e01867 (2014).

[12] Welch, P. The use of fast Fourier transform for the estimation of power spectra: a method based on time averaging over short, modified periodograms. *IEEE Trans. Audio Electroacoust.* **15**, 70-73 (1967).

[13] Fries, P. Rhythms for cognition: communication through coherence. *Neuron.* **88**, 220-235 (2015).

[14] Kingma, D. and Welling, M. Auto-Encoding Variational Bayes. *International Conference On Learning Representations (ICLR).* (2014).

[15] Friston, K., Mattout, J., Trujillo-Barreto, N., Ashburner, J. and Penny, W. Variational free energy and the Laplace approximation. *Neuroimage.* **34**, 220-234 (2007).
